# Single-cell heterogeneity underpins antagonistic antibiotic interactions

**DOI:** 10.1038/s44320-025-00163-9

**Published:** 2025-10-27

**Authors:** João Pedro Teuber Carvalho, Daniel Schultz

**Affiliations:** Department of Microbiology & Immunology – Geisel School of Medicine at Dartmouth, Hanover, NH 03755 USA

**Keywords:** Microbiology, Virology & Host Pathogen Interaction

## Abstract

J. Carvalho and D. Schultz discuss the study by Broughton et al, in this issue of *Molecular Systems Biology*, on the heterogeneous responses of individual *E. coli* to a combination of antibiotics, contributing to their antagonistic interaction.

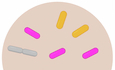

## Teaser

Using combinations of existing antibiotics is a promising strategy to treat bacterial infections. Although some drugs act synergistically, other drug combinations inhibit microbial growth less than what is expected from their individual effects. Ciprofloxacin and tetracycline display such antagonistic interaction. In a new study, Broughton and colleagues (Broughton et al, 2025) used single-cell microfluidics to show that exposing *E. coli* cells to a combination of ciprofloxacin and tetracycline results in highly heterogeneous outcomes. The survival of single cells is linked to the expression of moderate levels of the SOS response, which fixes the double-strand DNA breaks caused by ciprofloxacin. High expression of the SOS response was found only among dying cells. Tetracycline then counteracts ciprofloxacin by increasing the proportion of cells that survive treatment within the low-SOS subpopulation. These findings highlight the importance of single-cell studies in understanding the phenotypic heterogeneity that emerges during antibiotic responses, which decide the success of treatments.

**Figure 1:**
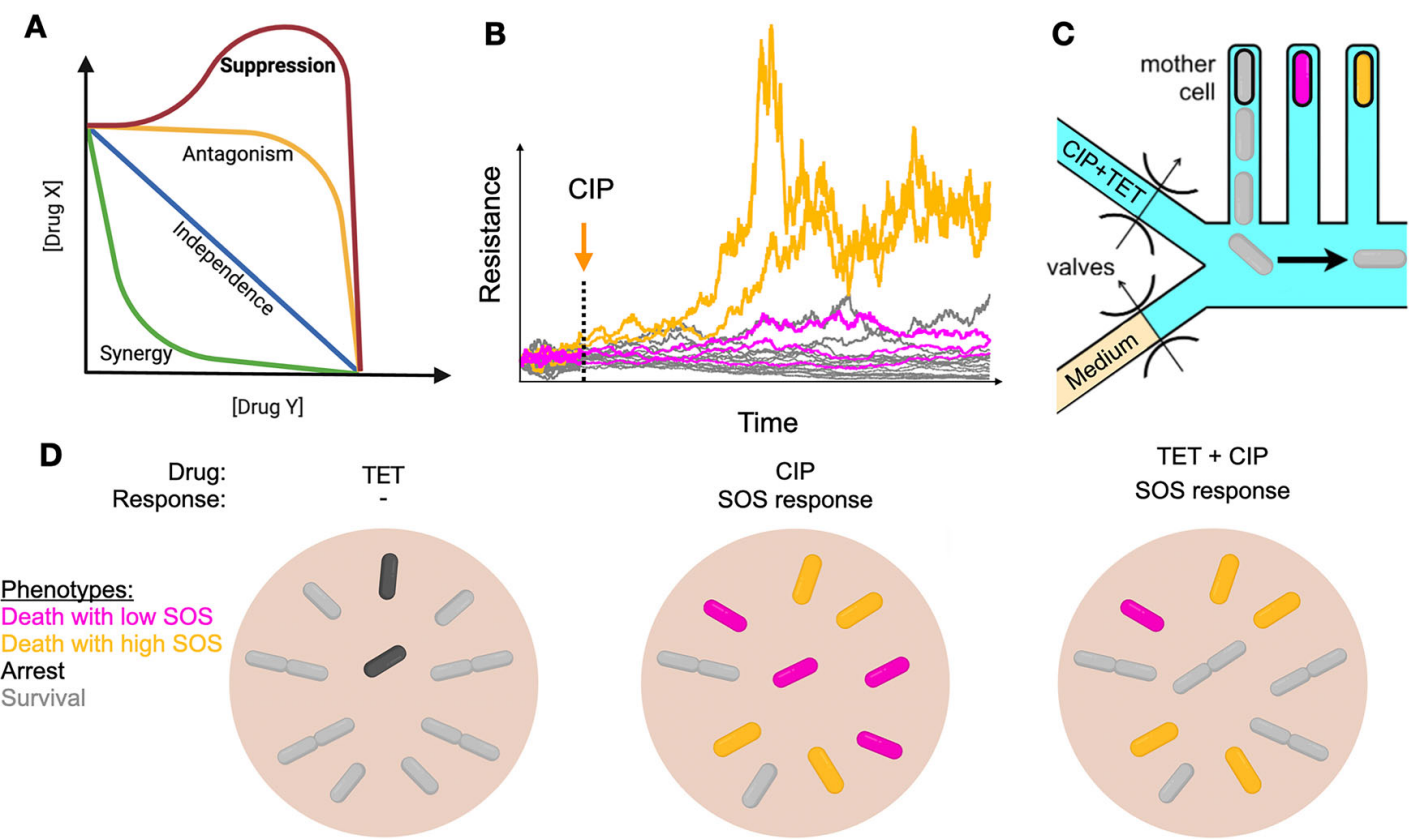
Phenotypic heterogeneity during responses to combined antibiotic treatment. (**A**) Antibiotics used in combination can interact by amplifying or reducing their action in comparison to what is expected from adding the effects of each individual drug. The type of interaction can be inferred from bulk cultures by the shape of the MIC isoboles with respect to each drug concentration. The combination ciprofloxacin (CIP) and tetracycline (TET) displays suppressive interactions, with tetracycline reducing killing by ciprofloxacin. (**B**) The induction of antibiotic responses, such as the SOS response that corrects the double-strand breaks caused by ciprofloxacin, is noisy due to stochasticity in gene expression. This noise can be amplified by feedback mechanisms resulting from the interplay between drug action, gene expression and cell metabolism, leading to phenotypic heterogeneity. (**C**) Single-cell microfluidics, such as the mother machine, allows direct observation of the emergence of phenotypic heterogeneity during antibiotic responses. Since these devices trap single bacteria before exposure to drug, they do not oversample from fast-growing lineages. (**D**) Distinct subpopulations during TET and CIP treatments, alone and in combination. In the concentrations used in this study, TET alone only inhibits cell growth. CIP alone results in heterogeneous cell fates, with noisy expression of the SOS response. When both antibiotics were used, tetracycline rescued cells within the low-SOS population, increasing the proportion of surviving cells.

The increased prevalence of antibiotic resistance in clinical pathogens, combined with the current difficulty in developing new drugs, creates an urgent need for creative strategies to more effectively employ the antibiotics that are currently available (Centers for Disease Control and Prevention, 2019; Clatworthy et al, 2007). Among these, the combination of multiple drugs to treat bacterial infections is a promising approach, being widely employed in the clinic today (Roemhild et al, 2022). By targeting multiple microbial cellular processes at once, drug combinations offer a broader spectrum of action and reduce the chance of resistance. However, due to the high connectivity of the cell environment, the cellular processes targeted by each of the antibiotics often exhibit unforeseen interactions (Fig. 1A). While some drugs act synergistically, reaching higher growth inhibition together than what is expected from adding the effects of each drug alone, other drug combinations have the counterintuitive effect of “canceling” each other, resulting in less effective treatments (Bollenbach et al, 2009). The mechanisms driving such antagonistic interactions are not yet well understood (Kavčič et al, 2020; Yeh et al, 2006).

Microbial survival to antibiotic treatments often depends on the activation of several responses, such as resistance mechanisms, stress responses, or metabolic shifts (Lopatkin et al, 2021). The noisy and dynamic process of inducing these responses upon drug exposures, which is tightly linked to the drug action on cell metabolism, has been shown to result in highly diverse outcomes among single cells (Deris et al, 2013; Stevanovic et al, 2024) (Fig. 1B). Single-cell microfluidics has been an essential tool in studying this emergence of phenotypic heterogeneity, allowing researchers to connect cell growth to the expression of relevant drug responses (Wang et al, 2010) (Fig. 1C). Even when only single drugs are used, noise in the expression of drug responses often results in the coexistence of live and dead cells, as well as intermediate slow-growing phenotypes with impaired cellular functions, which often die despite the expression of drug responses (Schultz et al, 2017). In such heterogeneous populations, at the edge of survival, slight differences in cellular metabolism can tip the balance of viability.

In a new study, Broughton and colleagues (Broughton et al, 2025) use single-cell microfluidics to study the interaction between antibiotics ciprofloxacin and tetracycline in *Escherichia coli*, showing not only that this combination creates heterogeneous phenotypes, but also that tetracycline counteracts ciprofloxacin by increasing the chances of single cells surviving the combined antibiotic exposure (Fig. 1D). Population-level studies have shown that when bactericidal antibiotics kill cells by targeting an active cellular process, slowing down cellular metabolism with a bacteriostatic drug can suppress this killing, leading to improved population growth. The fluoroquinolone ciprofloxacin targets type II topoisomerases, causing DNA double-stranded breaks that eventually lead to cell death. Tetracycline, in contrast, is a bacteriostatic that acts by targeting ribosomes, slowing down cellular metabolism. Indeed, population studies in liquid cultures have shown that these antibiotics interact antagonistically, with tetracycline reducing cell killing by ciprofloxacin.

Survival to ciprofloxacin treatments depends on the activation of the SOS response, which repairs the double-stranded breaks caused by the drug. Tracking the expression of the SOS response in single cells during exposures to ciprofloxacin, the authors found that, surprisingly, higher levels of SOS expression did not result in survival, with surviving cells typically expressing SOS at lower levels. Among the cells that died, SOS expression was bimodal, with cells expressing either high or low levels. Upon closer inspection, SOS expression was found to reach high levels only shortly before cell death. This observation is consistent with other studies where moribund cells with impaired functions accumulate high levels of response due to slow growth, highlighting the complexity of the interplay between drug action on cell metabolism and the expression of drug responses.

In this heterogeneous context, adding tetracycline to the ciprofloxacin treatment improves population growth by increasing the odds of survival of single cells. Although surviving cells grow slightly slower under the drug combination than with ciprofloxacin alone, this cost is offset by the reduced killing provided by the addition of tetracycline. Remarkably, the proportion of high-SOS dead cells is virtually not altered. Furthermore, the antagonistic effect of the drug combination depends on nutrient quality, with the richness of the medium decreasing survival under ciprofloxacin alone but increasing survival under the drug combination. Therefore, tetracycline rescues cells from ciprofloxacin death more effectively in fast-growing conditions.

Since pathogens colonize sites in the human host with varying nutrient availabilities and drug accessibilities, it is essential to understand the cellular mechanisms that underlie the interactions between different antibiotics and decide the fate of microbial infections. While considerable interest has been placed in finding synergistic combinations of drugs, studying antagonistic combinations is important for probing the couplings between the cell processes targeted by different antibiotics. Additionally, antagonistic interactions tend to slow or even prevent the evolution of antibiotic resistance by weakening selection pressures and creating complex fitness trade-offs, being potentially valuable for long-term management of resistance.

The remarkable coexistence of different phenotypes upon antibiotic exposures, even within isogenic populations in homogeneous environments, highlights the importance of single-cell studies to understand complex community behaviors. While population-level studies may be more practical, their bias towards fast-growing lineages masks the contribution of slow-growing subpopulations to antibiotic resistance. In particular, dissecting the dynamics of cellular processes that define the slow-growing moribund phenotypes hanging between life and death during antibiotic exposures will be crucial to understand what separates successful treatments from failures.
